# Correlation between clinical trial endpoints of marketed cancer drugs and reimbursement decisions in China

**DOI:** 10.3389/fpubh.2022.1062736

**Published:** 2022-11-24

**Authors:** Kexin Ling, Huli Qin, Yiman Feng, Hongxi Che, Jinxi Ding, Wei Li

**Affiliations:** ^1^School of International Pharmaceutical Business, China Pharmaceutical University, Nanjing, China; ^2^School of Pharmacy, China Pharmaceutical University, Nanjing, China; ^3^Pharmaceutical Market Access Policy Research Center, China Pharmaceutical University, Nanjing, China

**Keywords:** cancer drug, clinical trial endpoint, surrogate endpoint, drug price negotiation, drug reimbursement decision

## Abstract

**Objective:**

This study aimed to assess whether different clinical trial endpoints in pivotal trials of cancer drugs were associated with reimbursement decisions in China.

**Materials and methods:**

Cancer drugs marketed before June 30^th^, 2021 with publicly available technical review reports for application of drug registration on Center for Drug Evaluation (CDE) website were reviewed. The trial design characteristics and relevant clinical outcomes [e.g., overall survival (OS), progression-free survival (PFS) and objective response rate (ORR)] were extracted from the technical review reports, while the reimbursement decisions were reviewed from National Healthcare Security Administration (NHSA) website. The differences in trial characteristics and clinical outcomes between drugs with positive reimbursement decisions and negative ones were compared by hypothesis test (Pearson's chi-squared test, Fisher's exact test, independent samples *t*-test and Mann-Whitney U test). The correlation between different clinical trial endpoints and reimbursement decisions was analyzed by multivariate logistic regression.

**Results:**

There were 112 cancer drug indications included in this study. Among these indications, 76 received a positive reimbursement decision, and the most common primary endpoints of them were PFS (42.1%) and ORR (30.3%). Taking PFS (OR = 7.333) and ORR (OR = 5.271) as the primary endpoints were more likely to receive a positive reimbursement decision compared with OS (*P* = 0.003). The proportion of drugs marketed with phase I (75.0%) and phase II (85.7%) clinical trials receiving positive reimbursement decisions are significantly higher than those marketed with phase III clinical trials (61.3%, *P* = 0.043). The magnitude of clinical benefit only had subtle influences (P_risk benefit − OS_ = 0.627, P_risk benefit − PFS_ = 0.087, P_survival benefit − OS_ = 0.545, P_survival benefit − PFS_ = 0.189) on the drug reimbursement decisions, however, the drug prices and clinical needs also made a difference on that.

**Conclusion:**

This study found that, in Chinese drug price negotiations from 2017 to 2021, policymakers have focused more on meeting clinical needs and filling therapeutical gaps in National Reimbursement Drug List (NRDL), while requirements for the selection of primary endpoints, clinical trial phases, and clinical benefits have been reduced. In the future, emphasis should be put on the use of surrogate endpoints and clinical benefits.

## Introduction

Cancer is the main leading cause of death globally, with nearly 10 million people dying of cancer worldwide in 2020 (1). In China, cancer is also a serious health problem (2), and the latest statistics released by the National Cancer Center of China shown that there were about 4,064,000 new cancer cases (3) and 2,413,500 new cancer deaths in China in 2016 (4). The annual medical expenditure on cancer in China exceeded 220 billion yuan, and the average inpatient expenditure for lung and gastric cancer alone reached 25,000 yuan in 2020 according to *China Health Statistics Yearbook 2021*, imposing a heavy financial burden on patients (5).

Drug therapy is the primary means of cancer treatment (6). To promote the accessibility of cancer drugs, the Chinese government has launched five rounds of drug price negotiations from 2017 to 2021, and has released five editions of National Reimbursement Drug List (NRDL) since the first edition introduced in 2000 (7), including many clinically necessary but expensive exclusive cancer drugs into NRDL at reduced prices to expand drug coverage (8).

According to the concept of value-based strategic purchase of medical insurance (9), policymakers focus on assessing the clinical value of drugs, of which the core indicators are outcome and cost (10, 11). For outcomes, clinical trial endpoints are commonly used to measure clinical benefit, and are classified into clinical endpoint and surrogate endpoint based on whether they directly measure clinical benefit (12). Since overall survival (OS, defined as the time from randomization until death from any cause) can directly measure the survival outcome of patients, it is often used as the clinical endpoint for cancer drugs (12, 13). To shorten the duration of clinical trials and accelerate drug launch, investigators may choose surrogate endpoints related to survival benefit as clinical trial endpoints, such as progression-free survival (PFS, defined as the time from randomization until objective tumor progression or death, whichever occurs first) and objective response rate (ORR, defined as the proportion of patients with tumor size reduction of a predefined amount and for a minimum time period, equal to partial responses plus complete response) (12–14). On July 1^st^, 2020, China National Medical Products Administration (NMPA) permitted the application for drug marketing with surrogate endpoints through a conditional approval process (15, 16).

However, evidence shows unclear correlations between surrogate endpoints and OS, which means such surrogate endpoints may not accurately predict clinical benefit (17–20). Scholars are divided on drugs of uncertain clinical benefit, with some opposing the positive reimbursement decisions of drugs with unclear clinical benefits (21) and others advocating that a new standard for drugs with surrogate endpoints should be established (22). Previous studies have analyzed the relevance between reimbursement decisions and clinical trial endpoints (23–28), the situation is not consistent across countries, while few studies have focused on the situation in China.

This study systematically reviewed the clinical evidence of all marketed cancer drugs in China, explored whether the use of surrogate endpoints had an impact on reimbursement decisions, and further identified the correlation between clinical trial design, the clinical benefits and reimbursement decisions in China.

## Materials and methods

### Sample

All of our analysis were based on drug indications for the following reasons: firstly, drug registration evaluation was based on indications; secondly, although NRDL was managed based on drugs, not all indications of a drug could be reimbursed, one was that some drugs were only accessed part of indications due to payment restrictions, another was that some intra-list drugs had approved new indications after NRDL admissions. The above practice was more common for cancer drugs, usually with multiple indications, compared with other drugs.

Cancer drug indications included in this study were marketed before June 30^th^, 2021 and had publicly available technical review reports for application of drug registration (hereinafter referred to as “technical review reports”), including drugs treating solid tumors and hematologic malignancies. Data were collected until May 10^th^, 2022.

**Figure 1:**
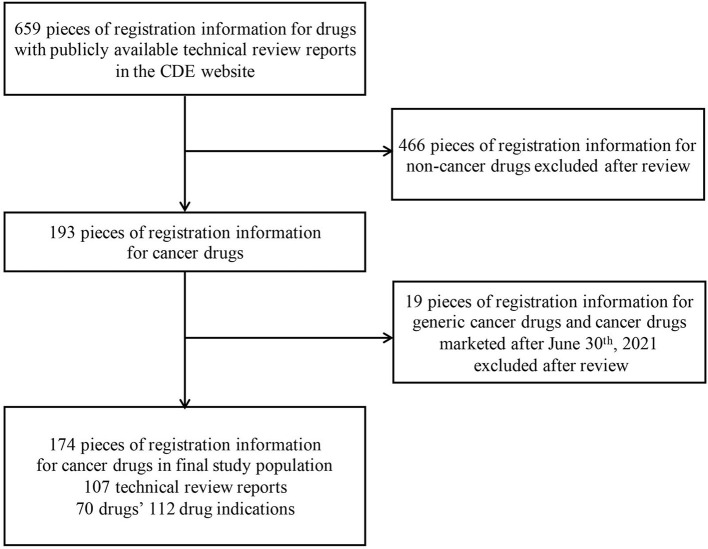
Identification process for eligible drug indications for analysis.

All the marketed drug information was exported from the Center for Drug Evaluation (CDE) website (29), and a total of 659 pieces of drug registration information with technical review reports were collected. We excluded registration information of non-cancer drugs, generic cancer drugs and cancer drugs marketed after June 30^th^, 2021, and got a total of 174 pieces of drug registration information (specific selection process was shown in Figure 1), which are corresponding to 70 drugs' 112 indications and 107 technical review reports.

In general, a drug indication corresponds to a technical review report. However, among the 107 technical review reports, 5 reports each included 2 indications, so the numbers of reports and indications were 107 and 112, respectively.

In addition, as some indications had multiple specifications, each specification corresponded to a piece of registration information, so the number of registered information was far more than the number of drug indications.

### Variables

A Microsoft Excel data form was created to extract the following variables: reimbursement decision, indication, tumor type, trial design characteristics (primary endpoint, phase, randomization, blinded trial, control group), and relevant clinical outcomes such as the hazard ratios (HRs) and the median OS or PFS. The clinical outcomes were used to describe clinical benefits. For the HRs, we defined “risk benefit” as HR < 1.0, which was equal to the value of 1 minus HR times 100%, meant drugs in the experimental group reduced the risk of death or disease progression. For the median survival time, we defined “survival benefit” as the difference in the median survival time in OS and PFS between the experiment and control groups, and calculated the median of the survival benefits in PFS and OS of drugs that received positive and negative reimbursement decisions separately. The definitions of variables were shown in Table 1.

**Table 1:** Research variables.

**Categories**	**Variables**	**Specific indicators**	**Definition**
Basic information	Indication	/	/
	Tumor type		
	Marketing time		
	Medical insurance access time		
Trial design characteristics	Primary endpoint	Only OS	Only taking one of OS, PFS and ORR as primary endpoint
		Only PFS	
		Only ORR	
		Other	Not taking any of the endpoints of OS, PFS, ORR as primary endpoint
		Two endpoints	Two endpoints in OS, PFS, and ORR are used simultaneously as primary endpoints
	Phase	Phase I	Phase of the pivotal clinical trials included in this study
		Phase II	
		Phase III	
	Blinded trial	Yes/No	Whether the pivotal clinical trials included in the study were blinded
	Control group	Yes/No	Whether the pivotal clinical trials included in the study had a control group
	Randomization	Yes/No	Whether the pivotal clinical trials included in the study were randomized
Clinical outcomes	Risk benefit	HR_OS_ data available (Yes/No)	Whether HR_OS_, HR_PFS_ data of the drug are provided
		HR_PFS_ data available(Yes/No)	
		Risk benefit in OS (Yes/No)	“Benefit” means an HR of < 1. “No benefit” refers to HR ≥ 1 or *P* ≥ 0.05 Risk benefit = (1-HR)100%
		Risk benefit in PFS (Yes/No)	
		Magnitude of Risk_OS_ benefit	Comparing HR values for drugs with risk-benefit, the smaller the HR, the greater the risk of disease progression or death reduced by the drug, and the greater the risk benefit.
		Magnitude of Risk_PFS_ benefit	
	Survival benefit	Survival benefit for mOS	The difference in median survival time between the experimental and control groups
		Survival benefit for mPFS	
	Reimbursement decisions	Positive /negative	Whether the drug indications are included in the NRDL (2021 edition)

### Data sources

The study extracted drug indications, corresponding tumor types, and time to market from the drug basic information module of the technical review reports, extracted clinical study design and clinical trial result data from the pivotal clinical trials of the technical review reports, extracted the reimbursement decisions from NRDL (2021 edition), extracted drugs failing the NRDL admission but applying for reimbursement from *the List of Drugs Passing the Preliminary Formal Review for Reimbursement Application* in 2020 and 2021; and extracted the time when drug indications first entered NRDL (2017 edition-2021 edition).

The inclusion criteria of the pivotal trial were: the clinical trial data of the Chinese population were preferentially used; if there was no clinical trial data of the Chinese population, the clinical trial results of the Asian population would be used; if there was none, the global clinical trial data would be used.

### Statistical analysis

Descriptive statistical analysis was conducted to describe tumor type, marketing time, and reimbursement time. Different methods were adapted based on the types of data to compare differences between groups of intra-NRDL drugs and extra-NRDL drugs in clinical trial design characteristics and clinical outcomes. For the count data, Pearson's chi-squared test and Fisher's exact test were used based on the sample size, to compare the trial design characteristics and clinical benefits with positive and negative reimbursement decisions. For the measurement data, independent samples *t*-test and Mann-Whitney U test were used according to whether they met normal distribution and homogeneity of variance, to test the difference in the magnitude of risk benefit and survival benefit with positive and negative decisions separately.

Statistically significant variables (*P* < 0.05) were included in the multivariate logistic regression analysis, and variables were selected by the maximum likelihood ratio-based forward stepwise method (α in = 0.05, α out = 0.1), with OR values and 95% confidence interval (CI) describing the degree of influence of the factors.

Data collecting and graphing were performed by Excel, and data analyzing was completed by SPSS, version 26.0.

## Results

### Characteristics of sample

Totally, 112 indications of 70 drugs approved from December 2014 to June 2021 with corresponding 107 technical review reports (among them, five reports containing two drug indications, respectively) were identified, among which 64 were new drug applications and 48 were new indication applications. Seventy-six received a positive reimbursement decision and 36 received a negative reimbursement decision. Of the relevant 112 clinical trials, 41 were global multicenter clinical trials, 17 were Asia Pacific clinical trials, and 54 were conducted in China.

The included drugs contain a total of 23 tumor types. Among them, non-small cell lung cancer (NSCLC) has the largest number of 20 (17.9%), followed by lymphoma (15, 13.4%) and breast cancer (11, 9.8%).

### Differences in trial design characteristics of drug with positive vs. negative decisions

There was no statistically significant difference in the distributions of blinded trial (*P* = 0.442), control group (*P* = 0.374), randomization (*P* = 0.453) between drugs that received positive and negative decisions. However, there were significant differences with regard to clinical trial phases (*P* = 0.043) and primary endpoints (*P* = 0.001), and the primary endpoint was the only statistically significant influencing factor in multi-factor logistic analysis (*P* = 0.003) (Tables 2, 3).

**Table 2:** Results of hypothesis testing.

**Categories**	**Variables**	**Options**	**Reimbursement decisions, No. (%)**		**Test**	***P*-value**
			**Positive (*n* = 76)**	**Negative** **(*n* = 36)**		
Trial design characteristics	Primary endpoint	Only OS	8 (10.5)	11 (30.6)	Pearson's chi-squared test	0.001
		Only PFS	32 (42.1)	6 (16.7)		
		Only ORR	23 (30.3)	6 (16.7)		
		Other	10 (13.2)	7 (19.4)		
		Two endpoints	3 (3.9)	6 (16.7)		
	Phases	I	3 (3.9)	1 (2.8)	Fisher's exact test	0.043
		II	24 (31.6)	4 (11.1)		
		III	49 (64.5)	31 (86.1)		
	Blinded experiment	Yes	28 (36.8)	16 (44.4)	Pearson's chi-squared test	0.442
		No	48 (63.2)	20 (55.6)		
	Control group	Yes	53 (69.7)	28 (77.8)	Pearson's chi-squared test	0.374
		No	23 (30.3)	8 (22.2)		
	Randomization	Yes	54 (71.1)	28 (77.8)	Pearson's chi-squared test	0.453
		No	22 (28.9)	8 (22.2)		
Clinical outcomes	HR_OS_ data availability	Yes	30 (39.5)	19 (52.8)	Pearson's chi-squared test	0.185
		No	46 (60.5)	17 (47.2)		
	Risk benefit in OS	Yes	17 (22.4)	18 (50.0)	Pearson's chi-squared test	0.011
		No	13 (17.1)	1 (2.8)		
		Missing	46 (60.5)	17 (47.2)	/	/
	Magnitude of risk benefit in OS	HR_OS_, median (interquartile range)	0.680 [0.530–0.747]	0.625 [0.428–0.743]	Two-sample *t* test	0.627
	HR_PFS_ data availability	Yes	41 (53.9)	18 (50.0)	Pearson's chi-squared test	0.696
		No	35 (46.1)	18 (50.0)		
	Risk benefit in PFS	Yes	37 (48.7)	16 (44.4)	Pearson's chi-squared test	1.000
		No	4 (5.3)	2 (5.6)		
		Missing	35 (46.1)	18 (50.0)	/	/
	Magnitude of risk benefit in PFS	HR_PFS_, median (interquartile range)	0.377 [0.280–0.563]	0.588 [0.280–0.735]	Two-sample t test	0.087
	Magnitude of survival benefit	Survival benefit for mOS, median	2.6 [1.875–5.875]	4.95 [1.430-6.680]	Mann-Whitney U Test	0.545
		Survival benefit for mPFS, median	5.00 [2.075–6.975]	2.10 [0.080–8.075]	Mann-Whitney U Test	0.189

**Table 3:** Results of multi-factor logistic analysis.

**Variables**	**B**	**S.E**.	**Wald**	**df**	**Sig**.	**Exp (B)**	**95% CI of EXP (B)**	
							**Lower**	**Upper**
Only OS			15.894	4	0.003			
Only PFS	1.992	0.643	9.593	1	0.002	7.333	2.078	25.875
Only ORR	1.662	0.653	6.485	1	0.011	5.271	1.466	18.944
Other	0.675	0.677	0.994	1	0.319	1.964	0.521	7.409
Two endpoints	−0.375	0.846	0.196	1	0.658	0.687	0.131	3.610
Constant	−0.318	0.465	0.470	1	0.493	0.727		

Of the 4 drugs marketed with clinical phase I studies, 3 (75.0%, 3/4) got a positive reimbursement decision; of the 28 drugs marketed with clinical phase II studies, 24 (85.7%, 24/28) received a positive reimbursement decision, while of the 80 drugs marketed with clinical phase III studies, only 49 (61.3%, 49/80) got a positive reimbursement decision.

The most common primary endpoints among the drugs that received a positive recommendation was PFS (42.1%) and ORR (30.3%). The proportion of drugs receiving a positive reimbursement decision choosing only OS (40.2%, 8/19) as the primary endpoint is lower than the figure for drugs choosing only PFS (84.2%, 32/38) or ORR (79.3%, 23/29). The results of multi-factor logistic regression analysis (Table 3) shown that compared with drugs taking OS as the primary endpoint only, drugs which took PFS (OR = 7.333) or ORR (OR = 5.271) as primary endpoint only were more likely to receive a positive reimbursement.

### Differences in clinical benefits of drugs with positive vs. negative decisions

Of the 112 drug indications, only 49 had HR_OS_ data available at the time of review. Of which 35 had showed risk_OS_ benefit, but only 17 received a positive reimbursement decision. 22.4% of drugs in NRDL showed risk benefit in OS while this proportion for drugs out of NRDL was 50.0%, which was a significant difference in risk_OS_ benefit between drugs in and out of NRDL (*P* = 0.011). Thirteen drugs without evidence of risk_OS_ benefit and 46 with no HR_OS_ data available also received a positive recommendation, which demonstrated clinical benefits in surrogate endpoints like PFS and ORR. The results shown that clinical benefit evidence was available for both drugs in and out of NRDL. Of the 112 drug indications, 59 had HS_PFS_ data available at the time of review. No significant differences were found in the distribution of drugs with evidence of risk_PFS_ benefit (*P* = 1.000) based on reimbursement decisions.

As for the magnitude of clinical benefit, no significant difference was observed, no matter risk benefit (P_riskbenefit − OS_ = 0.627, P_riskbenefit − PFS_ = 0.087) or survival benefit (P_survivalbenefit − OS_ = 0.545, P_survivalbenefit − PFS_ = 0.189).

**Figure 2:**
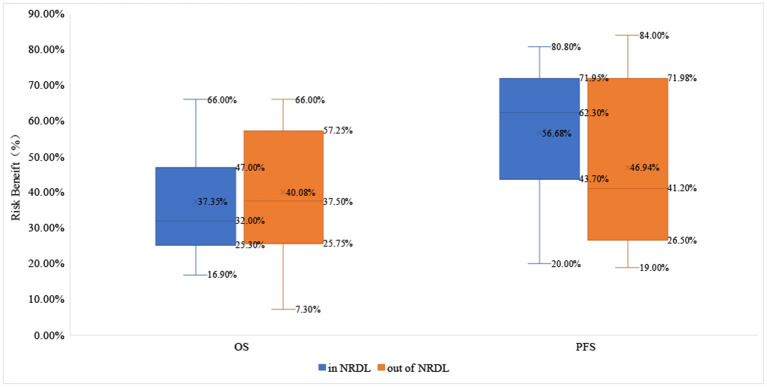
The risk benefit in OS and PFS of drugs in and out of NRDL.

**Figure 3:**
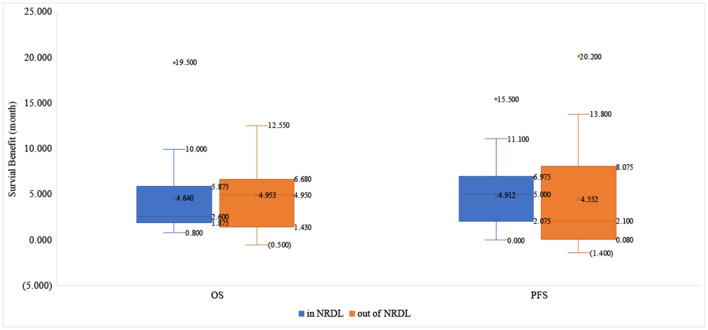
The survival benefit in OS and PFS of drugs in and out of NRDL.

About OS benefit, the drugs that received positive reimbursement decisions reduced the risk of death by 32.0% on average [HR_median_= 0.680 (0.530–0.747)], while the proportion for drugs that received negative reimbursement decisions is 37.5% [HR_median_= 0.625 (0.428–0.743)]. Similarly, the median survival benefit of drugs out of NRDL was higher than the proportion for drugs in NRDL [4.95 (1.430–6.680) vs. 2.60 (1.875–5.875)]. About PFS benefit, the drugs in NRDL and out of NRDL reduced the risk of death by 62.3% [HR_median_= 0.377 (0.280–0.563)] and 41.2% [HR_median_= 0.588 (0.280–0.735)], respectively, and the median survival benefit of drugs with positive vs. negative decisions was 5.00 (2.075–6.975) vs. 2.10 (0.080–8.075) (Table 2, Figures 2, 3).

## Discussion

### Impact of the primary endpoints selection on reimbursement decisions

The result showed that the primary endpoint selection was related to reimbursement decisions in China (Pearson's chi-squared test *P* = 0.001, multivariate logistic regression *P* = 0.003). The most common primary endpoints of drugs in NRDL were PFS (42.1%) and ORR (30.3%). Compared with OS, it was more likely to receive a positive reimbursement decision for drugs taking PFS (OR = 7.333) and ORR (OR = 5.271) as the primary endpoints.

Similar studies in other countries were examined to facilitate a qualitative comparison (24, 25), which shown that OS (92.6%) was the most common endpoint of drug that received a positive reimbursement decision, while only 7.4% of drug chose PFS in England and France. While in Canada, the most common primary endpoints with the positive reimbursement decisions were PFS (53.9%) and OS (32.1%), and the most frequently used endpoint for drugs with negative reimbursement decisions was ORR (38.5%) (Table 4).

**Table 4:** Study results of different nations.

**Indicators**		**China**	**Canada**	**England, France**
	Drugs with positive	OS:8 (10.5%);	OS:25 (32.1%);	OS:25 (92.6%);
	reimbursement decision	PFS:32 (42.1%);	PFS:42 (53.9%);	PFS:2 (7.4%);
		ORR:23 (30.3%);	ORR:5 (6.4%):	Other:0 (0%)
Selection of efficacy endpoints		Two endpoints:3 (3.9%);	Other:6 (7.7%)	
		Other:13 (13.2%)		
	Drugs with negative	OS:11 (30.6%);	OS:6 (23.1%);	OS:31 (83.8%);
	reimbursement decision	PFS:6 (16.7%);	PFS:6 (23.1%);	PFS:0 (0%);
		ORR:6 (16.7%);	ORR:10 (38.5%):	Other:6 (16.2%)
		Two endpoints:6 (16.7%)	Other:4 (15.4%)	
		Other:7 (19.4%)		
Association between endpoints selections and reimbursement decisions		Associated (*P* = 0.001)	Associated (*P* = 0.01)	No-associated (*P* = 0.991)

By sorting out the number of drugs and the tumor types included in the annual NRDL from 2017 to 2021 (7, 8, 30–36), the study found that the selection of primary endpoints associated with reimbursement decisions may be related to the China's drug reimbursement reform pace in recent years. Since 2017, China has conducted five rounds of price negotiations and adjustments of NRDL, and many cancer drugs successfully have gotten accessed to NRDL at significantly reduced prices. Of the 76 drugs included in this study, which comprised a total of 21 tumor types, 77.6% entered NRDL within 1 year of marketing, and only 3.9% entered NRDL 3 years after marketing.

The speed of incorporating cancer drugs in NRDL has been accelerating in recent years, and policymakers may focus more on filling treatment gaps in the list and meeting clinical needs, while slightly reduced the quality of clinical evidence. In this context, as OS usually requires a long follow-up time, pharmaceutical companies may prefer to take surrogate endpoints with shorter follow-up periods such as PFS to accelerate the launch and access of drugs in case of missing the policy window.

In addition, among the 11 drug indications taking OS as the primary endpoint but were not included in NRDL, two drugs (each corresponding to one indication), Gilteritinib and Radium Chloride [^223^Ra], were not included in *the List of Drugs Passing the Preliminary Formal Review for Reimbursement Application* and it was uncertain whether they applied for reimbursement (37); the seven indications corresponding to Nivolumab, Pembrolizumab and Ipilimumab, all of which were qualified for negotiation, were ultimately not included in NRDL due to high prices (38). It can be seen that taking OS as an endpoint was not the reason for their failure in NRDL admission, though it to some extent also influenced the results of the study (Table 5).

**Table 5:** List of drugs failed the NRDL admission by using OS as the primary endpoint.

**Generic name**	**Company**	**Indication**	**Date of marketing**
Gilteritinib fumarate tablets	Astellas Pharma Inc.	Acute myeloid leukemia	Jan. 30^th^, 2021
Radium [^223^Ra] chloride injection	Bayer AG	Castration- resistant prostate cancer	Aug. 26^th^, 2020
Trifluridine and tipiracil hydrochloride tablets	Taiho Pharmaceutical Co., Ltd.	Metastatic colorectal cancer	Aug. 29^th^, 2019
Venetoclax tablets	Abbvie Ireland Nl BV	Acute myelogenous leukemi	Dec. 2^nd^, 2020
Nivolumab injection	Ristol-Myers Squibb Holdings Pharma	Non-small-cell lung cancer	Jun. 15^th^, 2018
Nivolumab injection	Ristol-Myers Squibb Holdings Pharma	Squamous cell carcinoma of the head and neck	Sept. 29^th^, 2019
Nivolumab injection	Ristol-Myers Squibb Holdings Pharma	gastric cancer and adenocarcinoma of Esophagogastric junction	Mar. 12^th^, 2020
Nivolumab injection	Ristol-Myers Squibb Holdings Pharma	malignant pleural mesothelioma	Jun. 8^th^, 2021
Pembrolizumab injection	Merck Sharp and Dohme Corp., a subsidiary of Merck and Co., Inc.	Non-small-cell lung cancer	Sept. 29^th^, 2019
Pembrolizumab injection	Merck Sharp and Dohme Corp., a subsidiary of Merck & Co., Inc.	Esophageal squamous cell carcinoma	Jun. 17^th^, 2020
Ipilimumab injection	Bristol-Myers Squibb Pharma EEIG	Malignant pleural mesothelioma	Jun. 8^th^, 2021

### Impact of clinical trial phases on reimbursement decisions in China

Results of Fisher's exact showed that different phases of pivotal clinical trials were one of the influencing factors in drug reimbursement decisions (*P* = 0.043). The study found that the proportion of drugs having evidence from phase I (75.0%, 3/4) or II (85.7%, 24/28) studies entering NRDL is significantly higher than that of drugs with phase III (61.3%, 49/80). However, pan-Canadian Oncology Drug Review (pCODR) in Canada preferred to give positive reimbursement recommendations to drugs with phase III studies. If a phase III trial was deemed possible in later period, the drugs with phase II evidence was less likely to receive a positive recommendation from pCODR (*P* = 0.024) (26).

In order to explain the phenomenon mentioned above, we analyzed 31 drug indications that were marketed in Phase III clinical trials but were not in NRDL, but only found the reasons why 20 of them were not in NRDL (Table 6), and the remaining 11 drug indications did not find any disclosed news or information. It was found that the clinical needs of drugs marketed in Phase I and Phase II and those in Phase III are different, which may lead to a difference in the focus of policymakers when evaluating the drugs and ultimately affect the reimbursement decisions.

**Table 6:** Extra-list drugs with evidence from phase III clinical trial.

**Generic name**	**Indication**	**Date of marketing**	**Whether in the formal review list**	**The main reasons for negative reimbursement decision**
Olaparib tablets	Metastatic castration-resistant prostate cancer	Jun. 16^th^, 2021	Yes (2021)	Already in NRDL, applying for another indication, need to reduce price again
Osimertinib mesylate tablets	Non-small-cell lung cancer	Apr. 7^th^, 2021	No	Already in NRDL, applying for another indication, need to reduce price again
Lenvatinib mesilate capsules	Thyroid carcinoma	Nov. 4^th^, 2020	No	Already in NRDL, applying for another indication, need to reduce price again
Pertuzumab injection	Unresectable or metastatic HER2-low breast cancer	Dec. 6^th^, 2019	Yes (2021)	Competing products with the same indication in NRDL,such as Trastuzumab Emtansine for Injection; Already in NRDL, applying for another indication, need to reduce price again
Atezolizumab injection	Hepatocellular carcinoma	Oct. 28^th^, 2020	No	Competing products, Donafenib Tosilate Tablets and Lenvatinib Mesilate Capsules with the same indication in NRDL,
Enzalutamide soft capsules	Non-Metastatic Castration-Resistant Prostate Cancer	Nov. 2^nd^, 2020	No	Competing products, Apalutamide Tablets and Darolutamide Tablets with the same indication in NRDL;
Gilteritinib fumarate tablets	Acute myeloid leukemia	Jan. 30^th^, 2021	No	Competing product, Azacitidine for Injection, with the same indication in NRDL
Pralatrexate injection	Peripheral T cell lymphoma	Aug. 26^th^, 2020	Yes (2020, 2021)	Competing product, Trastuzumab Emtansine for Injection, with the same indication in NRDL
Venetoclax tablets	Acute myeloid leukemia	Dec. 2^nd^, 2020	Yes (2021)	Competing product, Azacitidine for Injection, with the same indication in NRDL,
Trastuzumab emtansine for injection	HER2-positive early breast cancer	Jan. 21^st^, 2020	Yes (2020)	Competing product, Azacitidine for Injection, with the same indication in NRDL
Palbociclib capsules	Receptor (HR)-positive, human epidermal growth factor receptor 2 (HER2)-negative advanced or metastatic breast cancer	Jul. 31^st^, 2018	Yes (2020, 2021)	Competing product, Abemaciclib Tablets, with the same indication in NRDL
Trifluridine and tipiracil hydrochloride tablets	Metastatic colorectal cancer	Aug. 29^th^, 2019	Yes (2020, 2021)	Competing products, Fruquintinib Capsules and Regorafenib Tablets, with the same indication in NRDL
Nivolumab injection	Gastric cancer and adenocarcinoma of esophagogastric junction	Mar. 12^th^, 2020	Yes (2020, 2021)	Competing product, Regorafenib Tablets, with the same indication in NRDL
Pembrolizumab injection	Squamous cell carcinoma of the head and neck	Dec. 8^th^, 2020	Yes (2021)	Competing product, Regorafenib Tablets, with the same indication in NRDL; Negotiations failed due to high price
Nivolumab injection	Malignant pleural mesothelioma	Jun. 8^th^, 2021	Yes (2021)	Negotiations failed due to high price
Pembrolizumab injection	Non-small-cell lung cancer^a^	Mar. 28^th^, 2019	Yes (2021)	Negotiations failed due to high price
Pembrolizumab injection	Non-small-cell lung cancer^b^	Sept. 29^th^, 2019	Yes (2021)	Negotiations failed due to high price
Pembrolizumab injection	Non-small-cell lung cancer^c^	Nov. 22^nd^, 2019	Yes (2021)	Negotiations failed due to high price
Pembrolizumab injection	Esophageal squamous cell carcinoma	Jun. 17^th^, 2020	Yes (2021)	Negotiations failed due to high price
Ipilimumab injection	Malignant pleural mesothelioma	Jun. 8^th^, 2021	Yes (2021)	Negotiations failed due to high price
Atezolizumab injection	Small cell lung cancer	Feb. 13^th^, 2020	Yes (2020)	Reasons not disclosed
Durvalumab injection	Non-small-cell lung cancer	Dec. 6^th^, 2019	Yes (2020,2021)	Reasons not disclosed
Lenalidomide capsules	Multiple myeloma	Dec. 20^th^, 2017	No	Reasons not disclosed
Lenalidomide capsules	Lymphoma	Nov. 17^th^, 2020	No	Reasons not disclosed
Nivolumab injection	Non-small cell lung cancer	Jun. 15^th^, 2018	Yes (2020,2021)	Reasons not disclosed
Nivolumab injection	Squamous cell carcinoma of neck	Sept. 29^th^, 2019	Yes (2020,2021)	Reasons not disclosed
Plerixafor injection	Lymphoma	Nov. 30^th^, 2018	Yes (2020)	Reasons not disclosed
Plerixafor injection	Multiple myeloma	Aug. 26^th^, 2020	Yes (2020)	Reasons not disclosed
Brentuximab vedotin for injection	Lymphoma	Apr. 13^th^, 2021	Yes (2020,2021)	Reasons not disclosed
Radium chloride [223Ra]	Prostatic cancer	Aug.26^th^, 2020	No	Reasons not disclosed
Blinatumomab for injection	Leukemia	Dec. 2^nd^, 2020	Yes (2021)	Reasons not disclosed

^a^ Pembrolizumab in combination with pemetrexed and platinum chemotherapy, is indicated for first-line treatment of patients with metastatic non-squamous NSCLC, with no EGFR or ALK genomic tumor aberrations.

^b^ Pembrolizumab is indicated as a single agent for the first-line treatment of patients with NSCLC expressing PD-L1 [Tumor Proportion Score (TPS) ≥1%] as determined by an NMPA-approved test, with no EGFR or ALK genomic tumor aberrations.

^c^ Pembrolizumab in combination with carboplatin and paclitaxel, is indicated for first-line treatment of patients with metastatic squamous NSCLC.

Most of the drugs with Phase I and Phase II marketing were for diseases that did not have effective treatments and were in urgent clinical need. To meet clinical demand, policymakers may loosen the restrictions on clinical trial evidence to make the drugs enter NRDL as soon as possible. Besides, among the 31 indications with evidence from phase III clinical trials but out of NRDL, 35.5% (11/31) of them already have the same indication treatment drugs in NRDL. In this case, policymakers will place more emphasis on the cost-effectiveness of the drugs instead of the quality of evidence, making NRDL access more difficult. In addition, 7 out of 31 drug indications, corresponding to 3 drugs, Nivolumab, Pembrolizumab and Ipilimumab, all failed to negotiate because of the disagreement on the price reduction; another four drugs (each corresponding to one indication), Olaparib, Osimertinib, Lenvatinib and Pertuzumab, has already been in NRDL, but adding the indications need to reduce prices again, which may affect the willingness of drug companies to negotiate (Table 6).

### Impact of clinical benefit on reimbursement decisions

The study shown that the difference in the magnitude of clinical benefits between drugs received positive and negative reimbursement decisions did not reach statistical significance (P_riskbenefit − OS_ = 0.627, P_riskbenefit − PFS_ = 0.087, P_survivalbenefit − OS_ = 0.545, P_survivalbenefit − PFS_ = 0.189). The clinical benefit of PFS was generally better than that of OS whether intra-list drugs or extra-list drugs. Furthermore, there is even a higher level of OS clinical benefit for drugs outside of NRDL than for drugs within NRDL.

One explanation for this was that the sample drugs were greatly influenced by price. Drugs such as Olaparib, Lenvatinib and Patuximab mentioned above, despite having OS clinical benefit, failed to negotiate due to the need for another price reduction for new indications. Some drugs, like Atelelizumab, Ipilimumab, Palivizumab and Nabrituzumab, were out of NDRL for their high prices. Another explanation was that more than 90% drugs with clinical benefits had evidence from phase III clinical trials, which meant it may not be clinically urgent. Under this situation, policymaker may pay more attention to cost-effectiveness ratio when making reimbursement decisions. It was shown that clinical benefit only has a weak impact on reimbursement decisions which needed to be considered in combination with the cost-effectiveness ratio of drugs and clinical needs.

### Limitations

This study had several limitations. First, we only included drugs with publicly available technical review reports, which resulted in small sample size. Second, since nearly 60% of the OS benefit data were missing, the results may be different from the total sample. Third, among the 36 drugs out of NRDL, 8 drugs weren't in *the List of Drugs Passing the Preliminary Formal Review for Reimbursement Application*, so we couldn't know whether they applied for reimbursement or not. Finally, drug reimbursement policy is a complex decision-making process, which should evaluate the effectiveness, safety, cost-effectiveness ratio, innovation, equity and other dimensions of drugs. While this study focused on the selection and improvement of efficacy endpoints, neglected indicators related to other drug evaluation dimensions and only included factors related to clinical trial design to minimize interference. So the impact of different drug evaluation dimensions on drug reimbursement policy will need to be further discussed in future studies.

## Conclusion

In Chinese drug price negotiations from 2017 to 2021, policymakers have focused more on meeting clinical needs and filling the therapeutic area gaps in NRDL, while requirements for the quality of clinical evidence (such as the selection of primary endpoints and clinical trial phases) and clinical benefits have been relaxed. It requires more attention to surrogate endpoints and clinical benefits of drugs.

For drugs with urgent clinical needs, the government should allow them apply for NRDL with surrogate endpoints and phase I or II clinical trials, however, it is necessary to continuously pay attention to the benefit of patients in the real world, and remove drugs that don't achieve the expected therapeutic effect promptly out of NRDL. For drugs that are not clinically urgent or there are other drugs with the same indications in NRDL, enterprises are encouraged to use OS endpoints and phase III clinical studies for NRDL application. For intra-list drugs with poor clinical outcomes and having extra-list competitors with better therapeutic effects, reevaluation should be adopted to include drugs with better efficacy in NRDL. Finally, a procedure for identifying surrogate endpoints should also be established, listing available surrogate endpoints for each disease type is necessary to regulate the use of surrogate endpoints in drug marketing and drug reimbursement policy.

## Data availability statement

The original contributions presented in the study are included in the article/supplementary material, further inquiries can be directed to the corresponding authors.

## Author contributions

KL contributed to study design and conception, data collection, data analysis, and manuscript drafting. WL and JD guided this study, including the design of the study, and the interpretation of data and the original draft preparation. HQ participated in the paper structure design, data verification, the manuscript drafting and modification. YF provided valuable suggestions for the thesis. HC provided great helps with figures depiction and revision. All authors contributed to the article and approved the submitted version.

## Funding

This work was supported by the Double First-class discipline innovation team construction project of China Pharmaceutical University (No. CPU2018GY4) and Quality social science application research project of Jiangsu Province in 2021 (No. 21SYB-096).

## Conflict of interest

The authors declare that the research was conducted in the absence of any commercial or financial relationships that could be construed as a potential conflict of interest.

## Publisher's note

All claims expressed in this article are solely those of the authors and do not necessarily represent those of their affiliated organizations, or those of the publisher, the editors and the reviewers. Any product that may be evaluated in this article, or claim that may be made by its manufacturer, is not guaranteed or endorsed by the publisher.
